# Manipulation of light spectrum can improve the performance of photosynthetic apparatus of strawberry plants growing under salt and alkalinity stress

**DOI:** 10.1371/journal.pone.0261585

**Published:** 2021-12-23

**Authors:** Majid Esmaeilizadeh, Mohammad Reza Malekzadeh Shamsabad, Hamid Reza Roosta, Piotr Dąbrowski, Marcin Rapacz, Andrzej Zieliński, Jacek Wróbel, Hazem M. Kalaji

**Affiliations:** 1 Department of Horticultural Sciences, Faculty of Agriculture, Vali-e-Asr University of Rafsanjan, Kerman, Iran; 2 Department of Horticultural Sciences, Faculty of Agriculture and Natural Resources, Arak University, Arak, Iran; 3 Department of Environmental Development, Institute of Environmental Engineering, Warsaw University of Life Sciences-SGGW, Warsaw, Poland; 4 Department of Plant Breeding, Physiology and Seed Science, Faculty of Agriculture and Economics, University of Agriculture in Krakow, Krakow, Poland; 5 Department of Bioengineering, West Pomeranian University of Technology in Szczecin, Szczecin, Poland; 6 Department of Plant Physiology, Institute of Biology, Warsaw University of Life Science, Warsaw, Poland; KGUT: Graduate University of Advanced Technology, ISLAMIC REPUBLIC OF IRAN

## Abstract

Strawberry is one of the plants sensitive to salt and alkalinity stress. Light quality affects plant growth and metabolic activities. However, there is no clear answer in the literature on how light can improve the performance of the photosynthetic apparatus of this species under salt and alkalinity stress. The aim of this work was to investigate the effects of different spectra of supplemental light on strawberry (cv. Camarosa) under salt and alkalinity stress conditions. Light spectra of blue (with peak 460 nm), red (with peak 660 nm), blue/red (1:3), white/yellow (1:1) (400–700 nm) and ambient light were used as control. There were three stress treatments: control (no stress), alkalinity (40 mM NaHCO_3_), and salinity (80 mM NaCl). Under stress conditions, red and red/blue light had a positive effect on CO_2_ assimilation. In addition, blue/red light increased intrinsic water use efficiency (*WUE*i) under both stress conditions. Salinity and alkalinity stress decreased OJIP curves compared to the control treatment. Blue light caused an increase in its in plants under salinity stress, and red and blue/red light caused an increase in its in plants under alkalinity. Both salt and alkalinity stress caused a significant reduction in photosystem II (PSII) performance indices and quantum yield parameters. Adjustment of light spectra, especially red light, increased these parameters. It can be concluded that the adverse effects of salt and alkalinity stress on photosynthesis can be partially alleviated by changing the light spectra.

## Introduction

Light is the plant’s primary energy supply, which stimulates various responses. It is a major environmental factor that affects the control of plant growth and development [1]. Plant growth and development need not only light with sufficient intensity, buy also quality [2]. Plants respond to changes in the quality of light by morphological, biochemical, and physiological changes. Such responses lead to an adjustment of the growth rate by changes in the environment’s energy availability [3]. Light-emitting diodes (LEDs) are used as radiation sources for plant planting systems and photobiological studies. The use of light-emitting diodes as a source of light has become common in agriculture [4]. The light produced by the LEDs led to improved fruit production and a large increase in strawberry yield. Increased photosynthesis (i.e., photosynthetic rate × period of photosynthesis) for high-yield strawberry production is essential [5]. The effects of different spectrum of complementary light on growing plants, nutritional content, and stress resistance have many practical horticultural consequences. Still, favorable outcomes, such as stress tolerance, resulting from applying a different spectrum of complementary lights, have not yet been shown. To better understand the mechanism of tolerance of plants to environmental stress in different light spectra, it is important to study not only the species, but also the varieties.

Plants are exposed to many abiotic stresses, and the most limiting factors for plant growth and development are salinity and alkaline stress [6]. Soil and water salinity and alkalinity are major abiotic stress for agriculture worldwide, causing severe harm to crop development and declining crop productivity [7]. Salinity stress blocks electron transfer from reaction centers (RCs) to plastoquinone [8]. It affects the electron transfer chain on the electron donor or acceptor side [9] and interfering with the electron transfer chain and reducing the efficiency of photosynthesis [10]. Together with the accumulation of salt in mesophyll cells, carbon uptake decreases, and the internal CO_2_ concentration increases, resulting in a decrease in stomatal conductance [11]. The most significant factor in reducing plant photosynthesis is stomatal closure under moderate and high salinity [12, 13]. Plant gas exchange characteristics are one of the most important criteria for studying stress tolerance in plants [14]. Through non-invasive measurement of photosynthesis, also chlorophyll fluorescence is more and more widely used. By this method, it is possible to determine the level of photosystem II (PSII) reaction centers damages caused by stress factor [15]. The research on the PSII efficiency of different plant species under stress conditions has been performed in the past. In response to stress conditions, these researches have reported a strong correlation between plant development and changes in the chlorophyll fluorescence parameters [16–19]. It has been also reported that the growth of strawberries under alkaline stress conditions decreases with decreasing chlorophyll content and photosynthetic performance index [18].

The most accurate analysis of the effect of a stressor on plants can be made by simultaneous measurements of gas exchange and chlorophyll *a* fluorescence [20]. In this way, the photochemical efficiency of plant photosynthesis can be analyzed and valuable information can be obtained about the components involved, especially PSII, in electron transfer in the photosynthesis process [21]. Photosystem II (PSII) is a part of the photosynthetic apparatus and is most sensitive to environmental stress. It plays a vital role in responding to the photosynthetic apparatus to stress conditions [22–26].

Much research has been done on the response of plants to light quality and its effect on photosynthesis, especially in the context of targeting emission spectra of LED sources to optimize horticultural production [27]. However, this type of studies should be extended with the possibility of improving of the photosynthetic apparatus efficiency of plants subjected to suboptimal environmental conditions with the use of artificial light sources. This may be especially important when the optimization of nutrition or growth substrate may be environmentally costly or even impossible, for example under organic production conditions. The aim of this work was to determining whether the negative impact of abiotic stresses on photosynthetic apparatus can be limited by different light spectra. We examined the effect of four different light variants on chlorophyll fluorescence and plant gas exchange parameters of strawberry cv. Camarosa under salinity and alkalinity stress. Salinity and alkalinity are stresses that inhibit plant growth, and much research has been done to reduce the effects of these stresses on plants. So, we hypothesize that different wavelengths of light affect plants’ response to these stresses by counteracting stress-induced imbalances in the functioning of the photosynthetic apparatus. This type of study is necessary to analyze the response of plants to lighting systems under stress conditions and to use LEDs with different wavelengths as radiation sources for plant research and horticultural production.

## Materials and methods

### Plant material and growth conditions

This experiment was conducted in the Vali-e-Asr University experimental greenhouse in 2020. The rooted strawberry plants (*Fragaria × ananassa* Duch, cV. Camarosa) were obtained from a nursery in Karaj, Iran. Plants were planted in a pot of 4 liters containing cocopeat and perlite (70:30). The plants were growing in a greenhouse with a temperature of 25/15±2°C (day/night), 11/13 h (light/dark) photoperiod, and relative humidity of 50±10%. During the growing period, irrigation of plants was done with Morgan nutrient solution [28] (EC: 1.4 dS m^-1^, pH: 6.5) (Table 1). Plants were treated by five light levels and three stress levels including: control (without stress), salinity (80 mM NaCl), and alkalinity (40 mM NaHCO_3_). To simulate salinity and alkalinity stresses under natural growth conditions (accumulation of salts during following growth stages) at first, salinity stress was induced by 40 mM NaCl and alkalinity by 20 mM NaHCO_3_. Then after, the concentrations of salt and alkalinity were increased until the effects of stress were observed on plants and the final concentrations reached 80 mM NaCl and 40 mM NaHCO_3_. The plants were stressed for 60 days and given NaCl and NaHCO_3_ every 4 days. Each treatment included three pots, and each pot had three plants.

**Table 1:** Concentration of nutrients used in the nutrient solution of this experiment.

Macronutrients	Concentration (mg L^-1^)	Micronutrients	Concentration (mg L^-1^)
**N**	128	Fe	5
**P**	58	Mn	2
**K**	211	Zn	0.25
**Ca**	104	B	0.7
**Mg**	40	Cu	0.07
**S**	54	Mo	0.05

### Light environments

**Fig 1:**
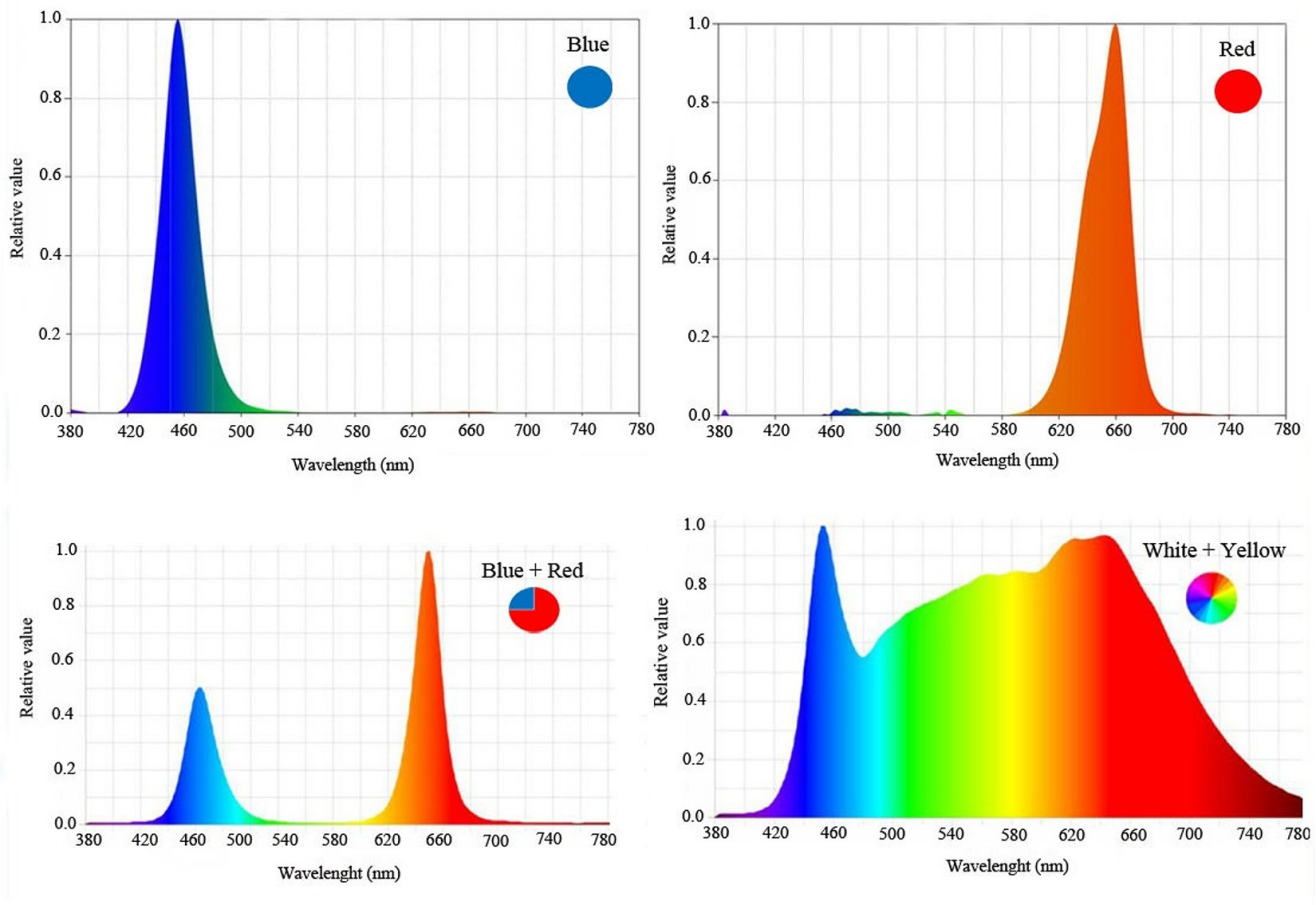
Relative distribution of different spectral LEDs (blue, red, blue/red (1:3) and white/ yellow (1:1) used during plant growth.

**Fig 2:**
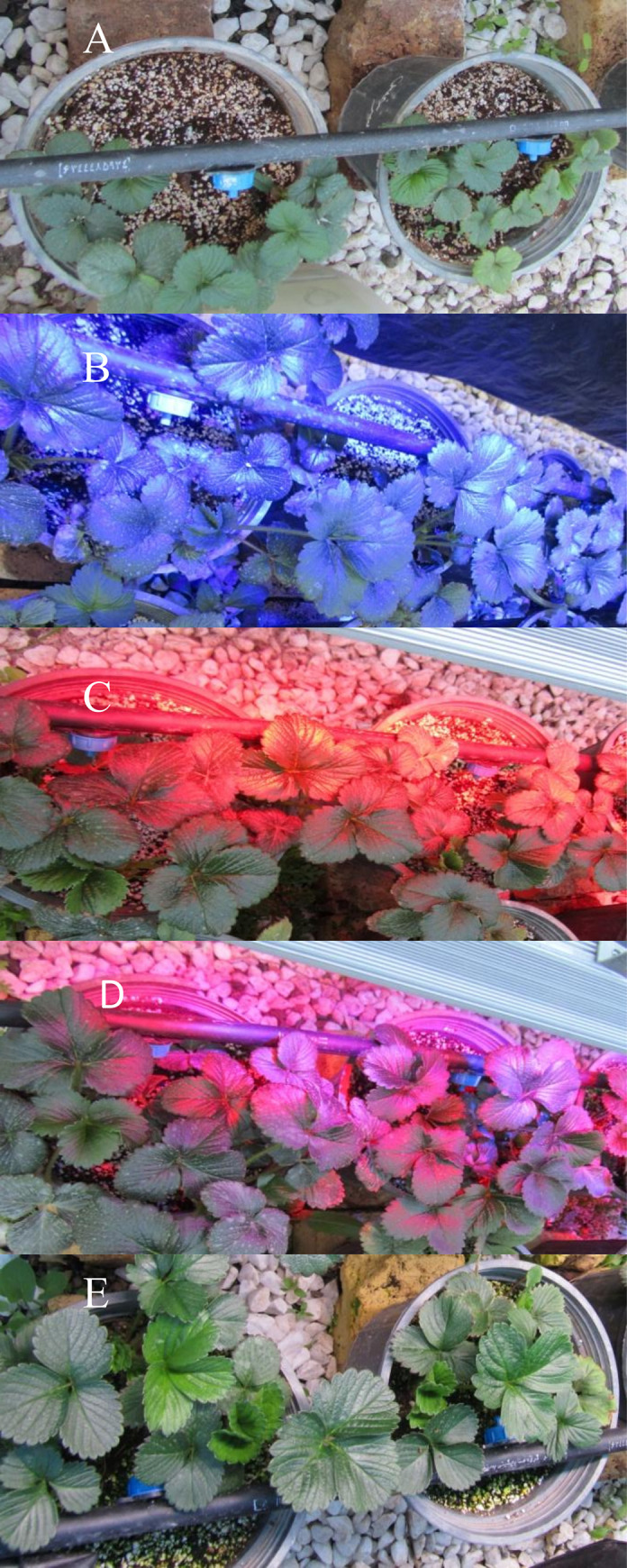
Strawberry plants (*Fragaria × ananassa* Duch, cV. Camarosa) under different light conditions (ambient light and different spectral LEDs: Blue, red, blue/red (1:3) and white/ yellow (1:1).

Plants cultivated under ambient light and under metal structures (length: 100 mm, width: 5mm, and height: 5 mm) with LED tubes with 24W of power (Parto Roshd Novin Company, Iran) (Table 2) of different spectral ranges: blue (B) (with peak 460 nm), red (R) (with peak 660 nm), blue/red (1:3), white LED combined with yellow (1:1) (400–700 nm) (Figs 1 and 2). Photon flux density (PPFD) was ca. 1000 μmol m^-2^ s^-1^ in all. The photoperiod of 11 hours was maintained. LED lighting systems were mounted 30 cm above each of the plant separately.

**Table 2:** Characteristics of LEDs used in this experiment.

manufacture company	CRI	Number of LEDs	light coverage area	Power consumption	Lens type	Certificate
Iran Grow Light	95%	24	40cm×100cm	24 watts	90°	CCC, CE, FEC, Ip45, RoHS

### Evaluation of chlorophyll fluorescence transient

The chlorophyll fluorescence parameters were measured by a portable photosynthetic analyzer 60 days after planting (PEA, Hansatech Instruments, King’s Lynn, UK). To measure, fully developed leaves adapted to a dark time for 15 min by fixing special clips on each upper leaf blade. Measurements were performed between 9:00 AM and 12:00 AM. The chlorophyll fluorescence measurements were carried out under greenhouse conditions, where the plants had grown. The saturating light (3,500 μmol m^-2^s^-1^ and 650 nm) induced transient of chlorophyll fluorescence extending from F_o_ to F_m_ (F_t_, fluorescence at time t after the start of the actinic lighting; F_o_ = F_30μs_, minimum fluorescence intensity; F_j_ = F_2ms_, fluorescence intensity at the J-step; F_i_ = F_30ms_, fluorescence intensity at the I-step; F_p_ = F_m_, maximum fluorescence intensity, at the peak P of OJIP) for all treatments. Based on the Strasser et al. method [8], the PSII parameters obtained from the OJIP transient were analyzed. Table 3 explains the parameters of chlorophyll fluorescence.

**Table 3:** The description of fluorescence parameters [8].

**Basic parameters derived from the extracted data**		
**F_o_**	Minimal fluorescence, when all PSII RCs are open	F_o_ = F20μs
**F_m_**	Maximal fluorescence, when all PSII RCs are closed	F_m_ (= FP)
**F_v_**	Maximal variable fluorescence	F_v_ = F_m_ - F_o_
**Area**	area between fluorescence curve and FM or Area above the fluorescence curve	
**normalized data**		
**F_v_/F_m_**	Maximum quantum yield of PSII	F_v_/F_m_ = φ_Po_ = TR_o_/ABS = [1 - (F_o_/F_m_)
**F_o_/F_m_**	A parameter related to changes in heat dissipation in the photosystem II antenna	1 - F_v_/F_m_
**F_v_/F_o_**	Efficiency of the water-splitting complex on the donor side of PSII.	(F_m_ - F_o_)/F_o_, 1/(1 - F_v_/F_m_) - 1, 1/(F_o_/F_m_) - 1
**V_j_**	Relative variable fluorescence at the J-step (t = 2ms)	(F_2ms_ - F_o_)/(F_m_ - F_o_)
**V_i_**	Relative variable fluorescence at time 30 ms (I-step) after start of actinic light pulse	V_I_ = (F_30ms_ - F_o_)/(F_m_ - F_o_)
**Specific energy fluxes (per active PSII reaction center)**		
**ABS/RC**	Absorption flux (of antenna Chls) per RC (also a measure of PSII apparent antenna size)	M_0_ (1/V_J_)(1/φ_Po_)
**DI_o_/RC**	Dissipated energy flux per RC at t = 0	ABS/RC - TR_o_/RC
**TR_o_/RC**	Maximal trapping rate of PSII	M_0_ = Vj
**ET_o_/RC**	electron transport in active RC	M_0_ × (1/V_J_) × ψ_0_
**RE_o_/RC**	Electron flux leading to the reduction of the PSI end acceptor	M_0_ × (1/V_J_)(1 - V_I_)
**performance indexes**		
**PI_ABS_**	Performance index for energy conservation from excitation to the reduction of intersystem electron acceptors	PI_ABS_ = (γ_RC_/1 - γ_RC_) (φ_Po_/1 - φ_Po_) (ψ_Eo_/1 - ψ_Eo_)
**PI_total_**	Performance index for energy conservation from excitation to the reduction of PSI end acceptors	PI_total_ = PI_ABS_ × δ_Ro_/(1 - δ_Ro_)
**The quantum yield for primary photochemistry**		
**φ_Po_**	Maximum quantum yield of primary PSII photochemistry (when all RCs are open, V = 0)	φP_o_ = TR_o_ /J_ABS_ = 1 - F_o_/F_m_
**Ψ_Eo_**	Efficiency/probability that an electron moves further than Q^A^-	ET_o_/TR_o_ = 1 - V_J_
**φ_Eo_**	Quantum yield for electron transport (ET)	ET_o_/ABS = (F_v_/F_m_) × (1 - V_J_)
**δ_Ro_**	Efficiency with which an electron from the intersystem electron carriers moves to reduce end electron acceptors at the PSI acceptor side (RE)	RE_o_/ET_o_ = (1 - V_I_)/(1 - V_J_)
**φ_Ro_**	It expresses the probability that an absorbed photon leads to a reduction of the PSI end acceptor	φ_Po_ + Ψ_Eo_ + δ_Ro_ = R_Eo_/ABS
**Slopes and integrals**		
**dVG/dt_o_**	Express the excitation energy transfer between the reaction centers	
**dV/dt_o_**	Express the rate of the reaction centers closure	
**S_m_**	Normalized area; it is related to the number of electron carriers per electron transport chain	S_m_ = Area/(F_m_ - F_o_) = Area/F_v_
**Leaf Gas Exchange**		
***A***	CO2 Assimilation Rate (μmol CO_2_ mol^-1^)	
***E***	Transpiration rate (mol m^-2^ s^-1^)	
***g*_s_**	stomatal conductance (mol H_2_O m^-2^ s ^-1^)	
***C*_i_**	Sub-stomatal CO_2_ concentration (μmol mol^-1^)	
***A*/*C*_i_**	instantaneous carboxylation efficiency	
**WUE_i_**	Instantaneous intrinsic water-use efficiency (μmol CO_2_ mol H_2_O^-1^)	

### Leaf gas exchange

Photosynthetic parameters include CO_2_ assimilation rate (*A*), stomatal conductance (*g*_s_), transpiration rate (*E*), sub-stomatal CO_2_ concentration (*C*_i_), instantaneous carboxylation efficiency (*A*/*C*_i_), and intrinsic water-use efficiency (*WUE*_i_) were measured 60 days after planting with portable photosynthesis system (ADC Bio Scientific Ltd, LCi-sd, Hoddesdon U.K.). Measurements were performed on fully grown leaves, between 9:00 AM and 12:00 AM. The air temperature and light intensity in cuvette during measurements were ambient. The CO_2_ concentration was 400 ppm.

### Experimental design and data analysis

The experiment was a complete randomized design with two factors in three replications in factorial form and three single-plant per pot. Using SAS software to analyze all data (SAS Institute, Cary, NC, USA). All data were statistically analyzed using two-way ANOVA model. When analysis of variance (ANOVA) indicated significant treatment effects, significant mean differences (P<0.05) were calculated by the LSD Multiple Range Test as a post hoc. Once the differences between the means are demonstrated, it is possible to determine which means are different using post hoc range tests and pairwise multiple comparisons. Range tests identify homogeneous subsets of means that do not differ from each other. The chlorophyll fluorescence parameters were calculated using the software "PEA Plus" version 1.12 (Hansatech). Pearson’s correlation coefficient was applied to determine the relationships among the parameters studied. The graphs were made using Excel 2013 (Microsoft, Redmont, WA, USA).

## Results

### Leaf gas exchange analyses

**Fig 3:**
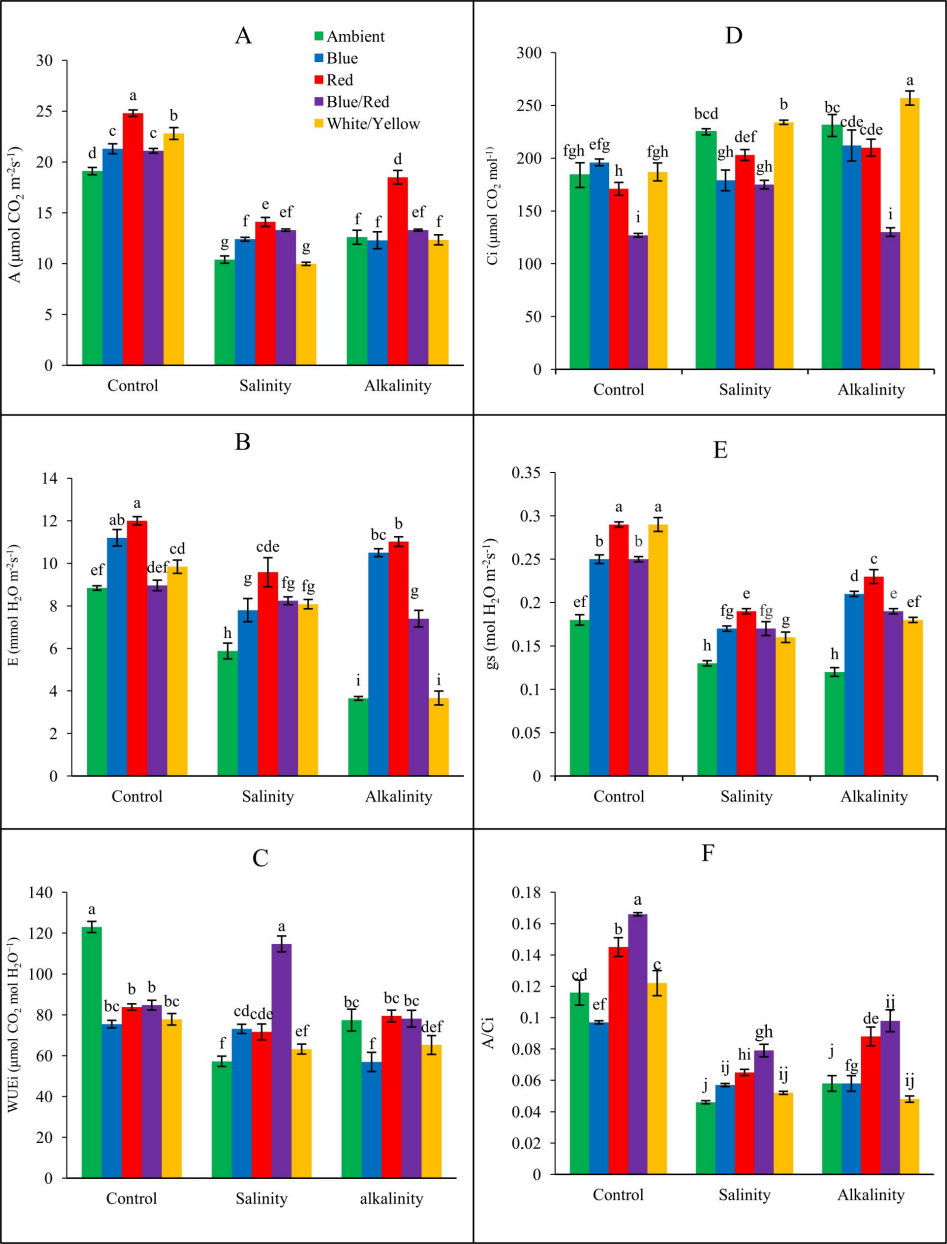
Leaf gas exchange parameters of different light spectra on strawberry cv. Camarosa under salinity and alkalinity stress conditions. A: CO_2_ assimilation rate (*A*); B: Transpiration rate (*E*); C: Intrinsic water-use efficiency (*WUE*i); D: sub-stomatal CO_2_ concentration (*C*i); E: Stomatal conductance (*g*_s_); F: Instantaneous carboxylation efficiency (*A*/*C*_i_). Means followed by the same letter for a parameter, are not significantly different according to the LSD (p ≤ 0.05). Vertical bars indicate the standard errors of three replicates.

Analysis of ANOVA (Table 4) shows different light spectra, stresses, and, what’s most important the interactions between them affect the gas exchange parameters of plants. CO_2_ assimilation rate (*A*) of plants was influenced considerably by salinity and alkalinity stress and different light spectra (Fig 3). CO_2_ assimilation rate decreased under stress conditions compared to the control. Red and red/blue light had a significant effect on increasing CO_2_ assimilation at salinity stress. In alkalinity stress, red light had a significant effect on the CO_2_ assimilation parameter, and other light treatments had no significant effect on CO_2_ assimilation.

**Table 4:** ANOVA results of different light spectra and stress on plant gas exchange parameters in strawberry cv. Camarosa.

Source of variations	Df	Means square					
		*C*_i_	*E*	*g*_s_	*A*	*A*/*C*_i_	*WUE*_i_
Light (L)	4	13933.68**	17.14**	0.0077**	89.899**	0.0063**	941.65**
Stress (S)	2	23451.28**	63.07**	0.0808**	581.14**	0.0396*	1245.2**
L × S	8	969.95**	6.826**	0.0034**	23.598**	0.0024**	103.02**
Error	30	98.93	0.392	0.00009	0.301	0.000016	10.49
CV%		4.8	8.27	4.98	4.42	5.79	5.25

**,*–significant at P≤0.01 and p≤0.05 respectively.

Parameters of stomatal conductance (*g*_s_) and transpiration rate (*E*) were also affected significantly by stress and different light spectra, and they decreased under stress conditions compared to the control. In salinity and alkalinity stress, red light had the most significant effect on increasing of these parameters compared to ambient light treatment. Under non-stress conditions, red and white/yellow light had a significant effect on *g*_s_. Also, in these conditions, red and blue light had a significant effect on *E*.

The internal CO_2_ concentration (*C*_i_) under stress conditions was significantly affected by applying different light spectra. Under both stress conditions, blue/red light had a significant effect on reducing this parameter. Blue light has also significant effect in plants under salinity stress. On the other hand, plants with white/yellow light treatment had the highest value of this parameter.

Salinity and alkaline stress decreased water use efficiency (*WUE*_i_) compared to the control. Water use efficiency was improved in the treatment with blue/red light in both stress conditions. Red light has also positively effect in plants under alkalinity stress. Salinity and alkaline stress reduced instantaneous carboxylation efficiency (*A*/*C*_i_) compared to the control. The highest instantaneous carboxylation efficiency in the control and salinity treatment was observed in blue/red light. In alkalinity stress, the highest *A*/*C*_i_ was observed in red and blue light treatment.

### Prompt chlorophyll *a* fluorescence

**Fig 4:**
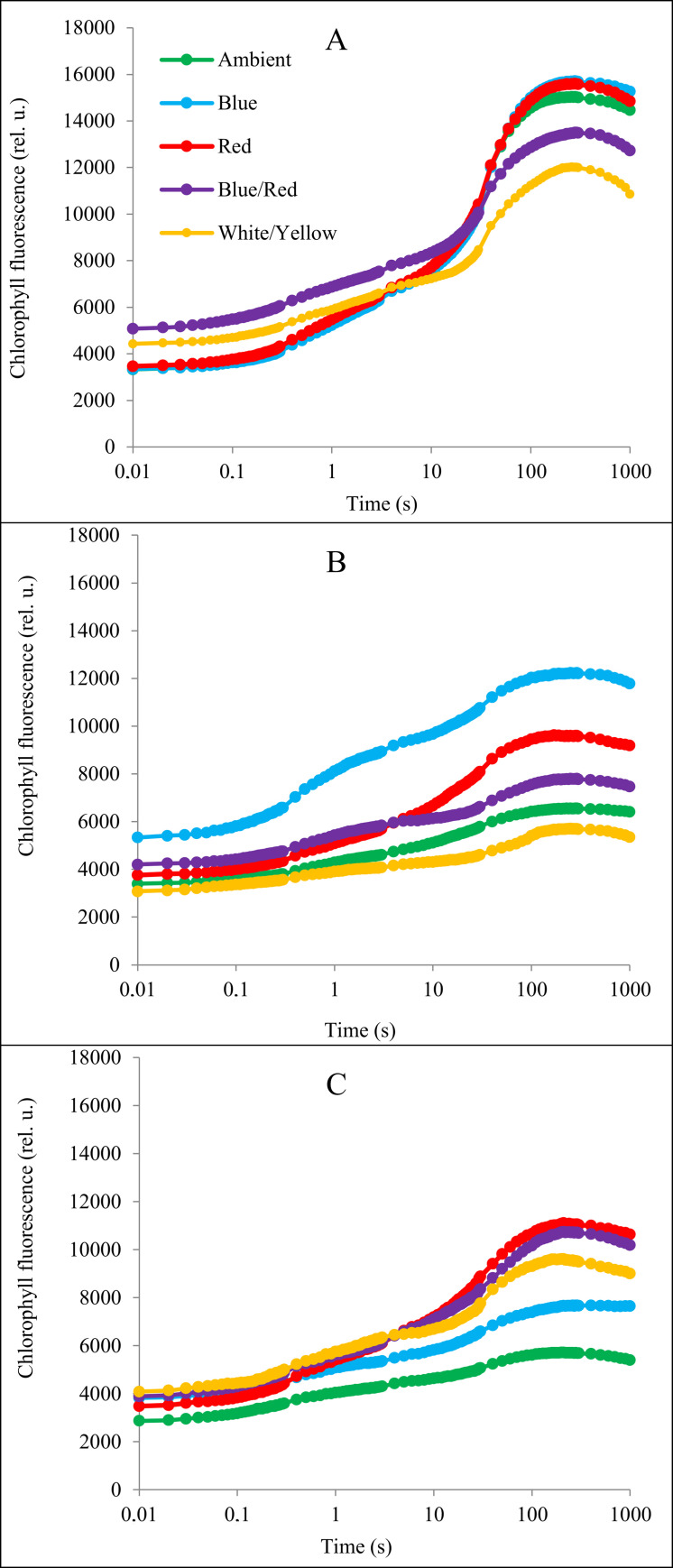
Induction curves of chlorophyll *a* fluorescence of strawberry cv. Camarosa grown under stress conditions and different light spectra. A: control conditions (without stress); B: salt stress: C: alkalinity stress.

Both, salinity and alkalinity stress significantly decreased the fluorescent transients compared to non-stresses plants in all light treatments, especially at the I and P steps. However, different light spectra reduced the stress effects and increased the fluorescent transients compared to the treatment without supplementary light. Under salt stress, the higher course in all points has the curve in plants with addition of blue light. In I and P points also addition of red light caused positively changes. The positively changes under alkalinity stress were only in I and P points and they were caused by red, blue/red and white/yellow light (Fig 4).

### Transients of chlorophyll fluorescent and calculated curves

**Fig 5:**
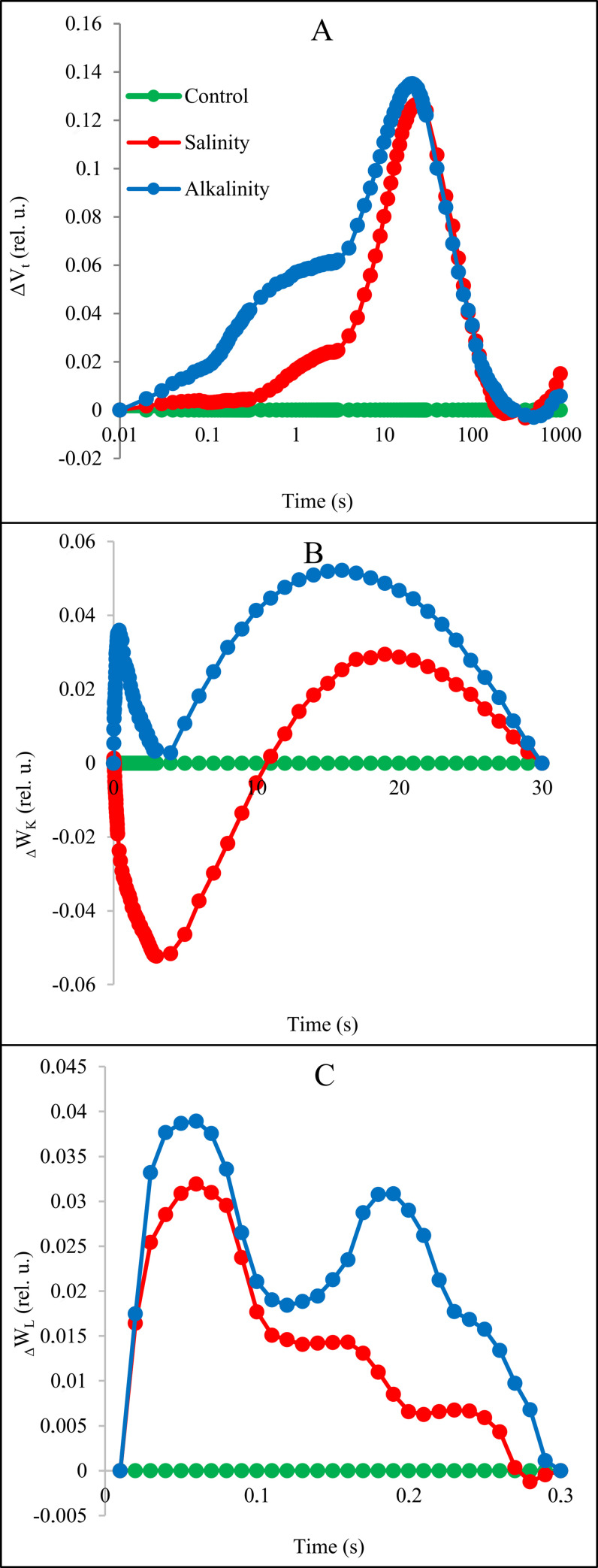
The effects of salinity and alkaline stress on chlorophyll fluorescence at ambient light conditions. A: differential curves of ΔV_t_; B: differential curves of ΔW_K_; C: Differential curves of ΔW_L_.

**Fig 6:**
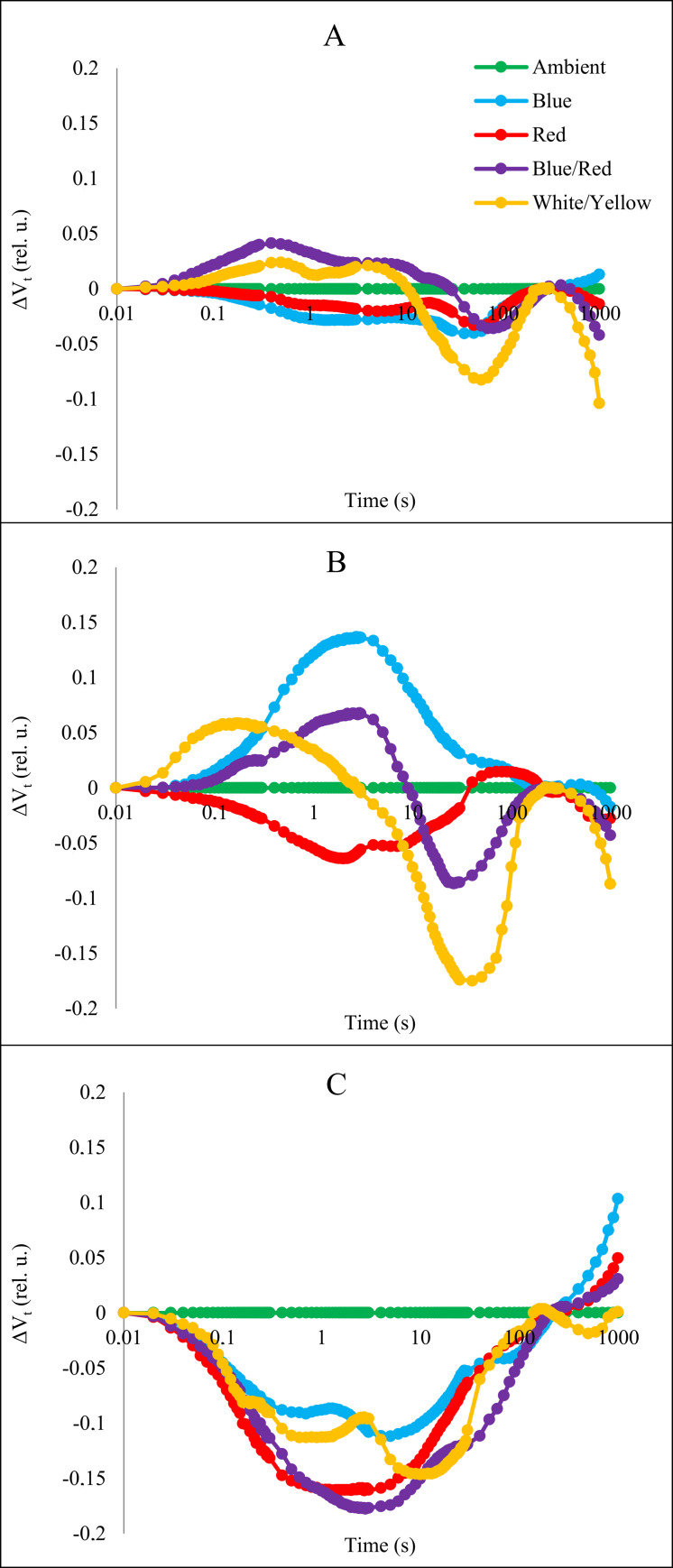
Differential curves of ΔV_t_ of chlorophyll *a* fluorescence of strawberry cv. Camarosa grown under stress conditions and different light spectra. A: without stress; B: salt stress: C: alkalinity stress.

The relative variable fluorescence curve was created to explore the effects of stress and light spectrum interaction on transient dynamics, V_t_ = (F_t_ - F_O_)/ (F_M_ - F_O_) [8]. Next, the changes in the OJIP fluorescence were calculated by subtracting the values of the fluorescence (O–P) recorded in plants under stress from those recorded for control plants (Figs 5 and 6). We observed significant changes in the prompt fluorescence of plants under salinity and alkaline stress at the J (V_J_) and I (V_I_) steps.

Based on these results, it can be concluded that different light spectra have significant effects in O−J and O−I stages under salinity and alkaline stress conditions. The positively effect in plants under salt stress was caused by blue light. In plants under alkalinity stress all light treatments caused negatively changes in curse of curves.

**Fig 7:**
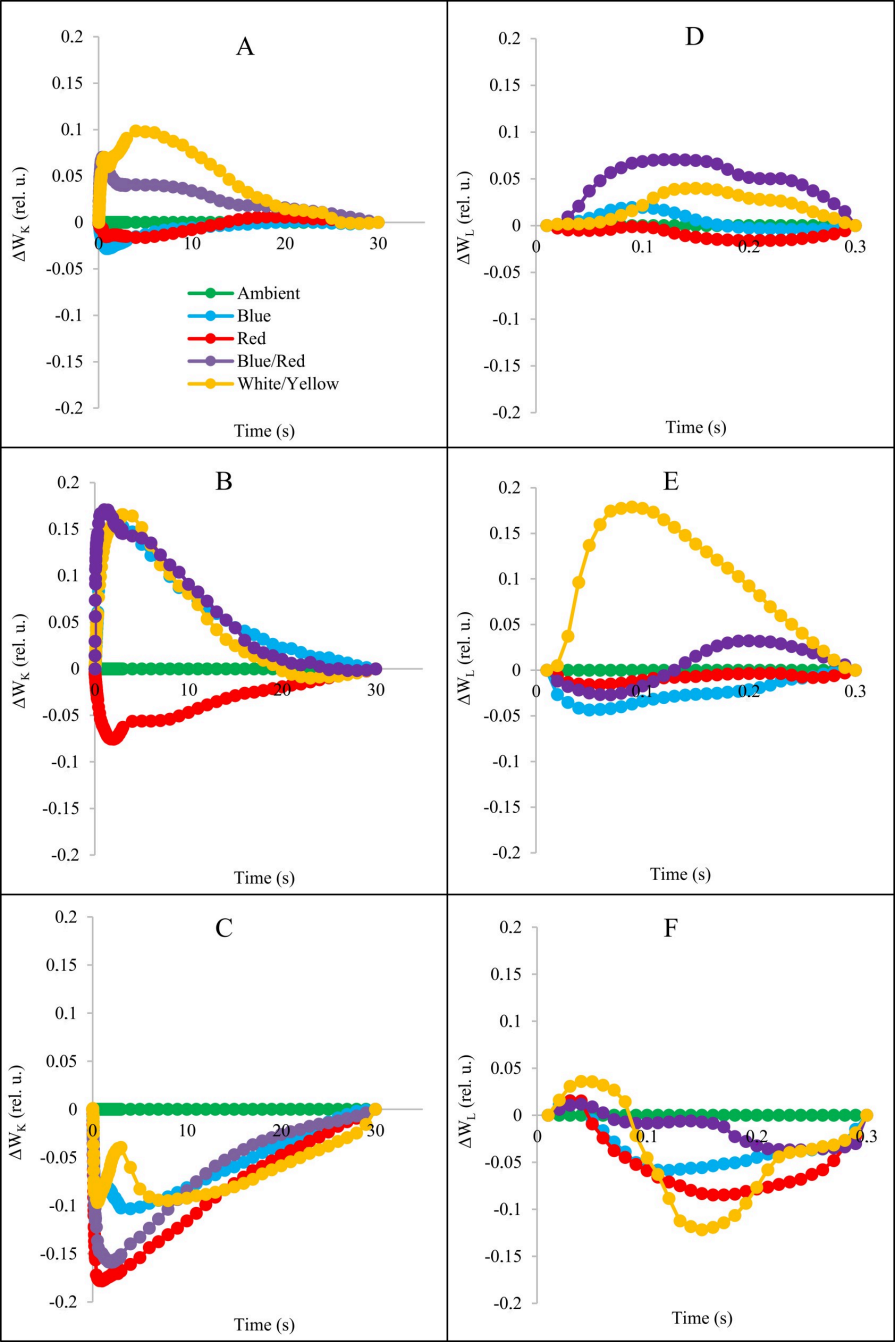
Differential curves of ΔW_K_ and ΔW_L_ under stress conditions and different light spectra. A: ΔW_K_ in control condition (without stress); B: ΔW_K_ in salt stress conditions; C: ΔW_K_ in alkalinity stress conditions; D: ΔW_L_ in control condition (without stress); E: ΔW_K_ in salt stress conditions; F: ΔW_K_ in alkalinity stress conditions.

For a detailed evaluation of stress conditions and changes in light spectra in OJIP fluorescence kinetics, we provide differential curves for the L and K bands that occur during the transient O to J. The curves of these bands were calculated by subtracting the amount of normal fluorescence (between O and K, O and J, respectively) recorded in the control plants from that recorded in the plants under stress and light spectra (Figs 5 and 7).

Stress conditions and different light spectra had a significant effect on ΔW_L_ and ΔW_K_ parameters. Salinity and alkalinity stress increased the L and K bands compared to the control treatment (Figs 5 and 7). The blue, blue/red and white/yellow light caused the largest increase in the K-band in plant treated by salt, and white/yellow light caused the largest increase in the L-band compared to other light spectra. In plants under alkalinity stress both, K-band and L-band decreased in all light treatments.

### JIP-test parameters

**Fig 8:**
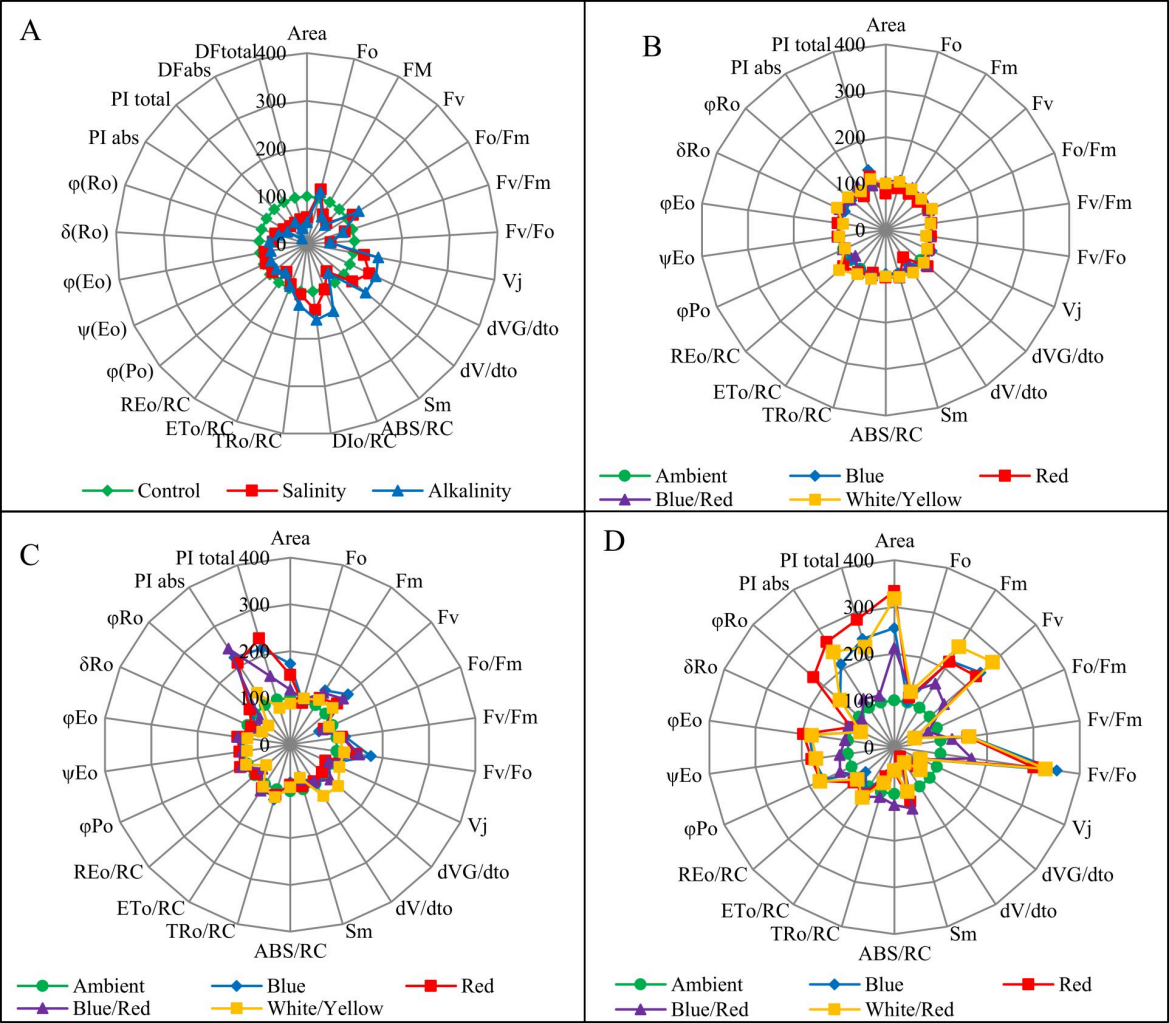
JIP-test parameters normalized on radar plots. A: the effects of salinity and alkaline stress at ambient light conditions; B: the effect of ambient light conditions on non-stressed plants; C: the effect of salt stress at different light conditions; D: the effect of alkalinity stress at different light conditions.

OJIP transients were converted into biophysical parameters: specific energy fluxes, performance indexes, quantum yield for primary photochemistry, and slopes and integrals [8]. The basic parameters derived from the extracted data were next normalized, which made it possible to compare the values measured in plants treated with LED light to plants treated with ambient light only. Salinity and alkalinity stress and different light spectra caused significant changes in these parameters compared to the control treatment (Fig 8 and S1 Table). Blue/red, blue and red light influenced positively on PI_ABS_ and PI_total_ parameters in plants under salt stress. Light with these spectra has also positively influence on F_v_ and F_v_/F_o_ parameters. Blue, white/yellow red light influenced positively the majority of parameters in plants under alkalinity stress: Area, F_m_, F_v_, F_v_/F_o_, φ_Po_, φ_Eo_, Ψ_Eo,_ PI_ABS_ and PI_total_. Moreover, Red light influenced φ_(Ro)_ parameter.

Analysis of ANOVA (Table 5) shows that different light spectra, stresses, and interactions affect the JIP test parameters. The stress treatments induced a decrease in F_m_ and increased F_O_ and, as a result, a decrease in F_V_. The use of light spectra increased F_m_ and F_v_. The performance indexes (PI_ABS_ and PI_total_) were significantly affected by treatments. Both stress treatments, especially alkalinity stress, caused a significant decrease in these parameters, and the use of light spectra, especially red light, increased these parameters compared to the control. Both stress treatments significantly reduced Quantum yield parameters (φ_Po_, φ_Eo_, φ_Ro_, Ψ_Eo_, δ_Ro_), and showed that these parameters are sensitive to environmental stresses. Different light spectra reduced the effects of stress and increased Quantum yield parameters compared to the control, and red light had the highest impact. Both stress treatments affected reaction centers, increased the ABS/RC, DI_o_/RC, and TR_o_/RC parameters. We observed that alkaline stress significantly increases the effective antenna size at each reaction center (ABS/RC). ET_o_/RC and especially RE_o_/RC decreased under salinity and alkalinity stress treatments, and red light had the most significant effect on these two parameters and increased them. The V_J_, dVG/dt_o_, and dV/dt_o_ parameters increased in plants treated by salt and NaHCO_3_, and red light reduced stress on V_J_ and dV/dt_o_ parameter more than other light spectra.

**Table 5:** ANOVA results of different light spectra and stress on JIP-test parameters in strawberry cv. Camarosa.

**Source of variations**	**Means square**												
	**Area**	**F**_**o**_	**F**_**m**_	**F**_**v**_	**F**_**o**_ **/F**_**m**_	**F**_**v**_ **/F**_**m**_	**F**_**v**_ **/F**_**o**_	**V**_**j**_	**dVG/dto**	**dV/dto**	**DF**_**abs**_	**DF**_**total**_	**S**_**m**_
Light (L)	**	**	**	**	**	**	*	**	**	**	**	**	**
Stress (S)	**	**	**	**	**	**	**	**	**	**	**	**	**
L × S	**	**	**	**	**	**	*	**	**	**	**	**	**
**Source of variations**	**Means square**												
	**ABS/RC**	**Dio/RC**	**TRo/RC**	**Eto/RC**	**REo/RC**	**φ**_**Po**_	**Ψ**_**Eo**_	**φ**_**Eo**_	**δ**_**Ro**_	**φ**_**Ro**_	**PI**_**ABS**_	**PI**_**total**_	
Light (L)	**	**	*	**	*	**	**	**	*	**	**	**	
Stress (S)	**	**	**	**	**	**	**	**	**	**	**	*	
L × S	**	**	**	**	**	**	**	**	**	**	**	**	

**,*–significant at P≤0.01 and p≤0.05 respectively.

### Correlation analysis

Based on the results, there is a significant relationship between some chlorophyll fluorescence parameters and the plant gas exchange parameter. Table 6 demonstrates the correlations between the plant gas exchange parameters (*A*, *WUE*_i_) and chlorophyll fluorescence parameters (F_v_/F_m,_ φ_Po,_ φ_Eo,_ φ_Ro,_ PI_ABS_ and PI_total_). The CO_2_ Assimilation Rate (*A*) had a positive correlation with F_v_/F_m_ (+0.58, p < 0.01)_,_ φ_Po_ (+0.40, p < 0.01)_,_ φ_Eo_ (+0.38, p < 0.01)_,_ φ_Ro_ (+0.69, p < 0.01)_,_ PI_ABS_ (+0.67, p < 0.01) and PI_total_ (+0.64, p < 0.01). The intrinsic water-use efficiency (*WUE*_i_) correlated positively with PI_ABS_ (+0.49, p < 0.01).

**Table 6:** Correlation coefficients between the plant gas exchange (*A*, *WUE*_i_) and chlorophyll *a* fluorescence parameters (F_v_/F_m,_ φ_(Po),_ φ_(Eo),_ φ_(Ro),_ PI_ABS_ and PI_total_).

	A	*WUE*_i_	F_v_/F_m_	φ_Po_	φ_Eo_	φ_Ro_	PI_ABS_
**A**	-						
***WUE*_i_**	0.47**	-					
**F_v_/F_m_**	0.58**	0.20^ns^	-				
**φ_Po_**	0.40**	0.13^ns^	0.86**	-			
**φ_Eo_**	0.38**	0.00^ns^	0.81**	0.9**	-		
**φ_Ro_**	0.69**	0.18^ns^	0.71**	0.66**	0.69**	-	
**PI_ABS_**	0.67**	0.49**	0.78**	0.75**	0.69**	0.72**	-
**PI_total_**	0.64**	0.17^ns^	0.65**	0.57**	0.56**	0.73**	0.64**

*, **, and ns: Significant correlation at the 0.05, 0.01 level, and non-significant, respectively. -, negative correlation, otherwise positive correlation.

## Discussion

The possibility of using LEDs as a light source is a major issue in terms of improving plant efficiency. The life cycle of plants is not only influenced by the intensity of light, but also by the composition of light spectrums. Therefore, in this work, we compared four different light spectra in terms of photosynthesis performance and chlorophyll *a* fluorescence parameters of strawberry cv. Camarosa under salinity and alkalinity stress conditions.

Numerous studies have shown that salt and alkaline stresses inhibit photosynthesis in different plant species [29, 30]. There is also demonstrated, that these stresses have a large effect on PSII [31, 32]. They affect photosynthetic parameters, which indicates that the photosynthetic apparatus is significantly sensitive to stresses [33]. However, there is a lack of knowledge, whether and how growth under different light spectrums can protect the PSII from salt and alkalinity stresses.

Our studies confirmed that salt stress affects CO_2_ assimilation. Moreover, we proved that both stresses effects on chlorophyll fluorescence. The stress of salinity and alkalinity affected both the parameters of F_o_ and F_m_. An increase in F_o_ and a decrease in F_m_ indicate a block in the transport of electrons from P680 to Q_A_ and the development of non-radiative dissipation of the excited states of PSII antennae chlorophyll [34]. A significant decrease in F_m_ indicates inhibition of electron flow in PSII and could be due to non-photochemical quenching, degradation of the D1 protein, or inactivation of RC PSII [11]. Kalaji et al., reported similar findings from the effect of salt stress on chlorophyll fluorescence [35]. A decrease in the maximum quantum yield of PSII (φ_Po_) shows that the stress conditions inhibit the redox reaction after Q_A_^-^ and slow the electron transport between Q_A_^-^ and Q_B_. A lower δ_Ro_ level indicates a decrease in electron outflow due to the deactivation of ferredoxin NADP^+^ -reductase in PSI [36]. Under salinity stress, PSII activity decreases due to harmful effects of salinity on manganese clusters, and also due to the separation of plastocyanin and cytochrome c553, PSI activity decreases [37]. In salt-stressed wheat leaves, PI_ABS_ decreased due to both ionic and osmotic stress [9], and the decline of PI_ABS_ was associated with a decrease in (F_m_ - F_J_)/F_v_ [38]. It has been reported that in alkaline conditions, Sm and PI index in strawberry plants has decreased [18]. Deng et al. reported similar results under salinity and alkalinity stress. The flux ratios ABS/RC, TR_o_/RC, and DI_o_/RC increased due to salinity and alkalinity stresses [39]. ABS/RC (total number of photons absorbed by Chl molecules) is obtained by dividing the total number of RCs by the total number of active RCs. As the number of inactive reaction centers increases, so does the ABS/RC ratio [40], which decreases the transport of electrons in active RC (ET_o_/RC) and reduces the final acceptor in PSI (RE_o_/RC). In plants grown in red, blue, and white/yellow LED light, electron transport flux per reaction center and the possibility that the trapped exciton will transfer the electron in the transport chain beyond the Q_A_^-^ (ψo) increased (Fig 6). The results showed that plants grown in red, blue, and white/yellow LED light could bring more electrons into the electron transport chain and beyond Q_A_ from absorbed photons, and this indicates that these plants after exposure to stress, positively regulate energy levels in the reaction centers [41].

According to our studies, changes in light spectra significantly affect the photosynthetic system of strawberry plants. These conclusions were in line with the results by Hogewoning et al., or Macedo et al., [42, 43]. We also found that under salinity stress, red and red/blue light had a positive effect on net-photosynthesis rate. In alkalinity stress, only red light partially mitigated the decrease in this parameter. Salinity and alkaline stress decreased water use efficiency (*WUE*_i_) compared to the control, but blue/red light had a significant positive effect on this parameter and maintain it at the control level under salinity. *WUE*i under salt stress was also improved by red and blue light used separately. In alkalinity stress, the highest A/C_i_ was observed in red and blue light treatment, and the other treatments had no significantly different from each other. The red and white/yellow light had the most significant effect on increasing fluorescence in plants under both stresses. Plants absorb mostly blue and red light (about 90%). Besides of absorption by photosystems blue light can indirectly affect stomatal opening which can be independent on photosynthetic activity (CO_2_ decrease in the leaf) and thus blue light can increase transpiration without significant effect on photosynthesis [44]. The opening of the stomatal with red light is due to the response of the stomatal guard cells to a decrease in the intercellular concentration of CO_2_ and the direct reaction of the chloroplast guard cells to red light [45]. Blue light is also essential for chlorophyll biosynthesis, and red light is also vital in this process [42]. The blue and red light combination is used in commercial research and horticulture because of the vital role of these wavelengths in photosynthesis. The lack of one of them (red or blue light) decreases the efficiency of photosynthesis. However, when the LED light is used as a complementary light in greenhouse lighting conditions, they may have different effects, and some wavelengths may be more effective.

The stomatal closure is the first defense of the plant against salinity and alkalinity stresses. Salinity affects photosynthesis by stomatal closure, reducing carbon uptake, and damaging photochemical reactions [46]. In stress conditions, and due to increased penetration resistance in stomatal and mesophilic cells, CO_2_ availability is affected [47], which reduces electron transfer to the final acceptor.

There is a high correlation between an increase in CO_2_ assimilation rate and an increase in PI_ABS_, PI_total,_ and quantum yield values, which provides evidence for changes in OJIP fluorescence rise kinetics using different light wavelengths under stress conditions with changes in the total capacity of photosynthesis. Under these conditions, the process of photosynthesis is regulated to maintain a balance between electron transfer reactions and carbon regeneration metabolism [48].

All the processes described above may point the very complex relationship between wavelength, environmental stresses and photosynthetic response. Additionally, studies on optimization of greenhouse light sources clearly pointed that the optimum light spectrum depends on plant species and developmental stage [49].

Our results indicate that another factor which can be considered in light optimization are potential abiotic stresses. The choose of optimum light conditions under stress may optimization of photosynthetic activity it is possible to balance the light phase energy production and the demand for this energy for carboxylation by the mentioned developmental effects and stomatal conductance. When the stress decreased energy demands the energy surplus in photosystems leads to damages or to reduction of long-term light phase capacity [50]. By changing light wavelengths and intensity we can regulate the energy supply to adjust them to the demand and also by manipulation of stomatal closure we can affect the demand and additionally water use, which may be also important for more sustainable agricultural production.

## Conclusions

The results of the present work suggest that the use of different light spectra can partially mitigate the deleterious effects of salinity and alkaline stress on photosynthesis of strawberry plants. Red and blue/red light had a significant effect on enhancing CO_2_ uptake under salinity stress conditions while under alkaline stress, only red light had a significant effect on its magnitude. We found that some chlorophyll fluorescence parameters can be used as bio-indicators to optimize the light spectrum for production systems that require a more resilient response to non-optimal growth substrate (too alkaline peat-free growth media) or nutrition (organic systems). Analysis of chlorophyll fluorescence parameters have demonstrated the changes in absorption, utilization, transmission, and dissipation of light energy by PSI and PSII in particular.

## Supporting information

S1 Table Descriptive statistics (minimum, maximum, means and S.D.) for gas exchange parameters used. (DOCX)
