# Supplementary material for: Manipulation of light spectrum can improve the performance of photosynthetic apparatus of strawberry plants growing under salt and alkalinity stress
Source: PLoS One. 2021 Dec 23;16(12):e0261585. doi: 10.1371/journal.pone.0261585 (PMC8699702; doi:10.1371/journal.pone.0261585)
Supplement: S1 Table — (DOCX) [file pone.0261585.s001.docx]

S1 Table. Descriptive statistics (minimum, maximum, means and S.D.) for gas exchange parameters used

| Light | Stress | Parameter | A | E | WUEi | Ci | gs | A/Ci |
| --- | --- | --- | --- | --- | --- | --- | --- | --- |
| Ambient light | Control | min/max | 18.6/20.1 | 8.6/9.0 | 119.4/128.4 | 161/199 | 0.18/0.19 | 0.107/0.133 |
|  |  | means | 19.11 | 8.8 | 123.0 | 184 | 0.18 | 0.116 |
|  |  | S.D. | 0.8 | 0.2 | 4.7 | 20 | 0.01 | 0.014 |
|  | Salinity | min/max | 9.9/11.2 | 5.3/6.6 | 54.0/62.2 | 220/230 | 0.13/0.14 | 0.044/0.048 |
|  |  | means | 10.48 | 5.8 | 57.2 | 225 | 0.14 | 0.046 |
|  |  | S.D. | 0.6 | 0.6 | 4.4 | 5 | 0.01 | 0.002 |
|  | Alkalinity | min/max | 12.5/12.8 | 3.5/3.8 | 66.8/84.2 | 206/257 | 0.12/0.13 | 0.052/0.069 |
|  |  | means | 12.7 | 3.6 | 77.4 | 231 | 0.12 | 0.058 |
|  |  | S.D. | 0.2 | 0.1 | 9.2 | 25 | 0.01 | 0.009 |
| Blue | Control | min/max | 20.6/21.5 | 10.7/12.01 | 71.7/77.4 | 191/202 | 0.24/0.26 | 0.095/0.099 |
|  |  | means | 21.3 | 11.2 | 75.4 | 196 | 0.25 | 0.097 |
|  |  | S.D. | 0.6 | 0.7 | 3.2 | 6 | 0.01 | 0.002 |
|  | Salinity | min/max | 12.2/12.7 | 6.7/8.5 | 69.0/76.0 | 162/196 | 0.17/0.18 | 0.055/0.059 |
|  |  | means | 12.4 | 7.8 | 73.2 | 179 | 0.17 | 0.057 |
|  |  | S.D. | 0.3 | 0.9 | 3.9 | 17 | 0.01 | 0.002 |
|  | Alkalinity | min/max | 10.9/13.8 | 10.2/10.7 | 49.9/65.9 | 206/220 | 0.21/0.22 | 0.049/0.067 |
|  |  | means | 12.3 | 10.5 | 56.9 | 212 | 0.21 | 0.058 |
|  |  | S.D. | 1.4 | 0.3 | 8.1 | 7 | 0.01 | 0.008 |
| Red | Control | min/max | 24.2/25.2 | 11.7/12.4 | 81.1/86.5 | 160/181 | 0.28/0.31 | 0.13/0.16 |
|  |  | means | 24.85 | 12.1 | 83.8 | 171 | 0.29 | 0.145 |
|  |  | S.D. | 0.5 | 0.3 | 2.6 | 10 | 0.01 | 0.011 |
|  | Salinity | min/max | 13.9/14.3 | 8.4/10.7 | 67.1/79.4 | 195/213 | 0.19/0.21 | 0.061/0.070 |
|  |  | means | 14.1 | 9.6 | 71.6 | 203 | 0.19 | 0.065 |
|  |  | S.D. | 0.2 | 1.2 | 6.7 | 9 | 0.01 | 0.004 |
|  | Alkalinity | min/max | 17.2/19.5 | 10.7/11.4 | 75.0/84.9 | 194/219 | 0.22/0.25 | 0.078/0.100 |
|  |  | means | 18.5 | 11.0 | 79.4 | 210 | 0.23 | 0.088 |
|  |  | S.D. | 1.1 | 0.4 | 5.0 | 14 | 0.02 | 0.011 |
| Blue/red | Control | min/max | 20.7/21.5 | 8.6/9.4 | 81.9/89.5 | 124/130 | 0.24/0.26 | 0.163/0.167 |
|  |  | means | 21.2 | 8.9 | 84.7 | 127 | 0.25 | 0.166 |
|  |  | S.D. | 0.4 | 0.4 | 4.1 | 3 | 0.01 | 0.002 |
|  | Salinity | min/max | 12.0/14.3 | 7.9/8.7 | 107.0/119.2 | 168/182 | 0.17/0.18 | 0.070/0.087 |
|  |  | means | 13.4 | 8.2 | 114.75 | 175 | 0.17 | 0.079 |
|  |  | S.D. | 1.2 | 0.4 | 6.7 | 7 | 0.01 | 0.008 |
|  | Alkalinity | min/max | 12.7/14.3 | 7.0/8.1 | 70.8/85.3 | 112/148 | 0.18/0.20 | 0.086/0.111 |
|  |  | means | 13.3 | 7.4 | 78.1 | 130 | 0.19 | 0.098 |
|  |  | S.D. | 0.8 | 0.6 | 7.2 | 18 | 0.01 | 0.012 |
| White/yellow | Control | min/max | 21.7/23.6 | 9.3/10.4 | 72.3/81.3 | 174/203 | 0.29/0.30 | 0.10/0.13 |
|  |  | means | 22.8 | 9.8 | 77.8 | 187 | 0.29 | 0.122 |
|  |  | S.D. | 1.0 | 0.5 | 4.9 | 15 | 0.01 | 0.014 |
|  | Salinity | min/max | 9.6/10.3 | 7.7/8.3 | 58.6/66.9 | 231/238 | 0.15/0.18 | 0.051/0.055 |
|  |  | means | 9.9 | 8.1 | 63.2 | 234 | 0.16 | 0.052 |
|  |  | S.D. | 0.3 | 0.3 | 4.2 | 3 | 0.01 | 0.001 |
|  | Alkalinity | min/max | 11.6/13.3 | 3.1/4.4 | 58.1/73.8 | 250/271 | 0.18/0.19 | 0.042/0.052 |
|  |  | means | 12.3 | 3.6 | 65.2 | 257 | 0.18 | 0.048 |
|  |  | S.D. | 0.8 | 0.6 | 7.9 | 11 | 0.01 | 0.004 |
